# The Impact of COVID-19 on the Delivery of Systemic Anti-Cancer Treatment at Guy’s Cancer Centre

**DOI:** 10.3390/cancers14020266

**Published:** 2022-01-06

**Authors:** Beth Russell, Charlotte Moss, Eirini Tsotra, Charalampos Gousis, Debra Josephs, Deborah Enting, Christina Karampera, Muhammad Khan, Jose Roca, Ailsa Sita-Lumsden, Kasia Owczarczyk, Harriet Wylie, Anna Haire, Daniel Smith, Kamarul Zaki, Angela Swampillai, Mary Lei, Vishal Manik, Vasiliki Michalarea, Rebecca Kristeleit, Anca Mera, Elinor Sawyer, Lucy Flanders, Irene De Francesco, Sophie Papa, Paul Ross, James Spicer, Bill Dann, Vikash Jogia, Nisha Shaunak, Hartmut Kristeleit, Anne Rigg, Ana Montes, Mieke Van Hemelrijck, Saoirse Dolly

**Affiliations:** 1 Translational Oncology and Urology Research (TOUR), School of Cancer and Pharmaceutical Sciences, King’s College London, London SE1 9RT, UK; charlotte.moss@kcl.ac.uk (C.M.); harriet.wylie@kcl.ac.uk (H.W.); Anna.haire@kcl.ac.uk (A.H.); Mieke.vanhemelrijck@kcl.ac.uk (M.V.H.); 2 Medical Oncology, Guy’s and St Thomas’ NHS Foundation Trust (GSTT), London SE1 9RT, UK; eirini.tsotra@gstt.nhs.uk (E.T.); harris.gousis@gstt.nhs.uk (C.G.); Debra.Josephs@gstt.nhs.uk (D.J.); Deborah.Enting@gstt.nhs.uk (D.E.); christina.Karampera@gstt.nhs.uk (C.K.); Muhammad.Khan@gstt.nhs.uk (M.K.); jose.roca@gstt.nhs.uk (J.R.); ailsa.lumsden@gstt.nhs.uk (A.S.-L.); Kamarul.Zaki@gstt.nhs.uk (K.Z.); vasi.michalarea@gstt.nhs.uk (V.M.); rebecca.kristeleit@gstt.nhs.uk (R.K.); lucyflanders@nhs.net (L.F.); sophie.e.papa@gstt.nhs.uk (S.P.); Paul.Ross@gstt.nhs.uk (P.R.); james.spicer@kcl.ac.uk (J.S.); hartmut.kristeleit@gstt.nhs.uk (H.K.); anne.rigg@gstt.nhs.uk (A.R.); ana.montes@gstt.nhs.uk (A.M.); Saoirse.dolly@gstt.nhs.uk (S.D.); 3 Clinical Oncology, Guy’s and St Thomas’ NHS Foundation Trust (GSTT), London SE1 9RT, UK; kasia.owczarczyk@gstt.nhs.uk (K.O.); daniel.smith@gstt.nhs.uk (D.S.); angela.swampillai@gstt.nhs.uk (A.S.); mary.lei@gstt.nhs.uk (M.L.); vishal.manik@gstt.nhs.uk (V.M.); elinor.sawyer@gstt.nhs.uk (E.S.); irene.defrancesco@gstt.nhs.uk (I.D.F.); 4 Guy’s and St Thomas’ NHS Foundation Trust (GSTT), London SE1 9RT, UK; anca.mera@gstt.nhs.uk (A.M.); bill.dann@gstt.nhs.uk (B.D.); vikash.jogia@gstt.nhs.uk (V.J.); 5 Immunoengineering Group, King’s College London, London SE1 9RT, UK; 6 Pharmacy, Guy’s and St Thomas’ NHS Foundation Trust (GSTT), London SE1 9RT, UK; nisha.shaunak@gstt.nhs.uk

**Keywords:** COVID-19, systemic anti-cancer treatment, oncology

## Abstract

**Simple Summary:**

Early reports during the pandemic stated that cancer patients were at increased risk of severe COVID-19 infection and mortality than non-cancer patients. However, delaying cancer treatment can cause worsening symptoms and shorten life expectancy. This study aimed to assess the outcome of cancer patients at our centre to help inform the optimal way to deliver cancer treatment during the COVID-19 pandemic. This study of over 4000 patients found that the delivery of cancer treatment in the first wave of the pandemic did not have a significantly negative effect on mortality. There were low COVID-19 infection (2%) and death rates (6%). As expected, there was a reduction in cancer treatments mainly in later lines of palliative treatments. This likely reflects patient and clinician choice around those more vulnerable patients. This data can be used to re-assure the continuation of cancer treatment during the pandemic.

**Abstract:**

Background: This study aimed to assess the outcome of cancer patients undergoing systemic anti-cancer treatment (SACT) at our centre to help inform future clinical decision-making around SACT during the COVID-19 pandemic. Methods: Patients receiving at least one episode of SACT for solid tumours at Guy’s Cancer Centre between 1 March and 31 May 2020 and the same period in 2019 were included in the study. Data were collected on demographics, tumour type/stage, treatment type (chemotherapy, immunotherapy, biological-targeted) and SARS-CoV2 infection. Results: A total of 2120 patients received SACT in 2020, compared to 2449 in 2019 (13% decrease). From 2019 to 2020, there was an increase in stage IV disease (62% vs. 72%), decrease in chemotherapy (42% vs. 34%), increase in immunotherapy (6% vs. 10%), but similar rates of biologically targeted treatments (37% vs. 38%). There was a significant increase in 1st and 2nd line treatments in 2020 (68% vs. 81%; *p* < 0.0001) and reduction in 3rd and subsequent lines (26% vs. 15%; *p* = 0.004) compared to 2019. Of the 2020 cohort, 2% patients developed SARS-CoV2 infections. Conclusions: These real-world data from a tertiary Cancer Centre suggest that despite the challenges faced due to the COVID-19 pandemic, SACT was able to be continued without any significant effects on the mortality of solid-tumour patients. There was a low rate (2%) of SARS-CoV-2 infection which is comparable to the 1.4%-point prevalence in our total cancer population.

## 1. Introduction

When the SARS-CoV2 outbreak was declared a pandemic in March 2020, there were major concerns as to whether cancer patients were at a higher risk of developing severe disease compared to the rest of the population. During the early stages, the true risk of COVID-19-related mortality for cancer patients was unknown with initial reports from China reporting high mortality and infection rates in lung cancer patients particularly [1]. As patients with cancer often have a compromised immune system, many cancer patients were advised to shield during the first lockdown in the UK. There were however, no data to quantify the additional risk of potentially immunosuppressive anti-cancer treatments, particularly chemotherapy, for these cancer patients. It was unknown whether by continuing systemic anti-cancer treatments (SACT), these vulnerable patients would be put at an additional risk due to increasing their potential exposure to SARS-CoV2 infection through frequent visits to the high-risk hospital setting. Due to the lack of national guidelines early in the pandemic, at our centre, decisions around treatment were made on an individual basis and were based around a subjective weighing up of risks of comorbidities, cancer stage, treatment paradigm and patient wishes.

Over the past year, there have been emerging data for cancer patients globally. The rate of infection among cancer patients appears to be significantly higher than in the general population. Furthermore, studies, including ours have identified risk factors for severe disease and COVID-19-related death in cancer patients including males, those of Black or Asian ethnicity and patients with haematological malignancies [2]. There have however, been conflicting results as to whether patients on active anti-cancer treatment are at an increased risk of COVID-19 and COVID-19-related death. Furthermore, there is a paucity of published large-scale data of SACT and COVID-19. Moreover, there are several collaborations of datasets across countries and healthcare systems, however, these tend to focus on COVID-19 positive patients only. Hence, there is a need to assess real world granular data inclusive of both COVID-19 positive and negative patients.

Therefore, this study aimed to determine whether cancer patients on SACT were at higher risk of developing COVID-19 disease and death using a comparator population of patients treated before the pandemic. Our primary objective was to compare the characteristics and mortality of patients treated with SACT during the pandemic in 2020 to those treated pre-pandemic in 2019. Secondary objectives were to delve deeper into the characteristics of the COVID-19 positive patients. This overarching aim was to identify factors which could help inform clinical decisions around COVID-19 enforced changes in standard treatment.

## 2. Materials and Methods

### 2.1. Study Population

Guy’s Cancer is one of the largest comprehensive Cancer Centres in the UK, treating approximately 8800 patients across South-East London per year including 4500 newly diagnosed patients. We were at the centre of the first wave of the UK COVID-19 epidemic in 2020. A database was established in which we prospectively evaluated all cancer patients with SARS-CoV2 infection, defined as a positive RT-PCR test, as previously described [2]. Herein, we analysed data on cancer patients who received SACT from 1 March to 31 May 2020 and compared this with patients treated over the same period in 2019. The COVID-19 database was then used to access data for those who were COVID-19 positive. All data were collected and analysed as part of Guy’s Cancer Cohort (Ethics Reference number: 18/NW/0297).

From our SACT electronic prescribing system, we extracted a list of solid tumour patients who received at least one treatment episode over the three-month period during the pandemic (2020) compared to the same period of 2019. This also included patients undergoing treatment at Guy’s Centre and Queen Mary Hospital, Sidcup. Patients with haematological malignancies and those that did not receive treatment were excluded. The clinical data were checked for accuracy by oncologists. SACT was defined as any prescribed anti-cancer treatment be it chemotherapy (CT), immunotherapy (IO), biological or targeted treatments (BT) or combined modalities. Biological treatments included biologics such as monoclonal antibodies and interferon. Targeted treatments were small molecule inhibitors, endocrine treatment for breast and prostate, somatostatin analogues and bisphosphonates. We collected data on patient demographics: age, gender, ethnicity, socioeconomic status (SES) and performance status (PS). Cancer characteristics including cancer type and stage. SACT details: modality and paradigm neo/adjuvant, radical or palliative and stage of palliative treatment. For the pandemic cohort treated in 2020, COVID-19 outcomes were collected: SARS-CoV2 infection defined as a positive RT-PCR test, severity by WHO criteria (mild, moderate, severe or death). We also collected overall mortality with data lock on the 1 March 2021. Mortality data were obtained through the electronic medical records from the five network hospitals, as well as updates from local acute oncology service and then cross-checked with death rates from monthly National imports. Given the close network working, we anticipate the loss-to-follow-up to be minimal.

### 2.2. Statistical Analyses

Data were analysed in a descriptive manner. Demographic and clinical characteristics were compared between the two years of study, 2020 and 2019, using T tests for continuous variables and Chi-squared tests for categorical variables. Tests of proportions were used to refine the Chi-squared results when needed. Characteristics of the COVID-19 positive SACT patients were described and were stratified by SACT type e.g., CT, IO, BT and combined treatments.

Multinomial logistic regression models were conducted to produce relative risk ratios (RRR) when considering year of treatment (2019 vs. 2020) as the exposure and SACT type, treatment paradigm and tumour stage as the dependent variable. Models were adjusted for age, sex, ethnicity and SES. Due to different follow-up periods for the 2019 and 2020 cohorts, a logistic regression model was conducted to estimate the relative risk of 6-month mortality in 2020 vs. 2019. Models were adjusted for age, sex, ethnicity, SES, cancer type, cancer stage and treatment paradigm. Sensitivity analyses were carried out whereby only patients with stage IV disease were included. This analysis was stratified by those on lines 1 and 2 palliative treatment. Models in the sensitivity analyses were adjusted for age, sex, ethnicity, SES, and cancer type.

All data management and analyses were carried out using StataIC 15.1 (College Station, TX, USA).

## 3. Results

### 3.1. Cohort Demographics

A total of 4569 patients with solid tumours received SACT within this retrospective study (Table 1). 2120 received SACT during the first wave of the UK COVID epidemic in March-May 2020 compared to 2449 in 2019 (13% decrease). Demographics were comparable between the two periods and as expected for an urban London population. Mean age was 61.8 (SD 13.0) during COVID-19 period in 2020 compared to 62.5 (SD 13.2) pre pandemic 2019 group. The majority of patients were female (56% vs. 54%) and of low SES (85% vs. 83%) in 2020 vs. 2019, respectively. Caucasian ethnicity was the most common (*n* = 831, 39% in 2020 vs. *n* = 1171, 48% in 2019). Patients from a Black, Asian minority ethnic (BAME) backgrounds represented similar proportions in 2019 (14%) and 2020 (13%).

### 3.2. Cancer and SACT Characteristics

The most common tumour type was breast with 24% in 2019 and 27% in 2020, followed by GI (23% in 2019 and 20% in 2020) and urological (22% in both 2019 and 2020) (Table 1).

There was an increase in the proportion of patients being treated with SACT for stage IV disease from 2019 to 2020 (62% to 72%, *p* < 0.0001) (Table 1). In the multinomial logistic regression analysis, there was however no clear pattern observed as patients in 2020 were more likely to be treated for stage II and IV disease (RRR = 1.44, 95% CI: 1.01–2.05 and RRR = 1.89, 95% CI: 1.40–257 respectively), but not for stage II disease (RRR = 1.11, 95% CI: 0.80–1.54) relative to stage I disease (Table 2).

There was, however, a decrease in chemotherapy (42% vs. 34%, *p* = 0.001), a slight increase in IO though not significant (6% vs. 10%, *p* = 0.245) and similar rates of biological/targeted treatments (37% vs. 38%) (Table 1). In 2020, the relative risk of patients receiving immunotherapy, relative to chemotherapy, was higher than those treated in 2019 (RRR = 1.69, 95% CI: 1.35–2.11) (Table 2). A similar association was observed for biological/targeted treatments (RRR = 1.27, 95% CI: 1.11–1.45) and for combined biological/targeted treatments (RRR = 2.30, 95% CI: 1.82–2.92).

Treatment paradigms were similar in 2019 and 2020: neo/adjuvant (29% vs. 28%), radical (4% vs. 1%) and palliative (67% vs. 69%) (Table 1). In terms of relative risk, patients treated in 2020 compared to 2019 were less likely to be treated with palliative (RRR = 0.39, 95% CI: 0.24–0.62), radical (RRR = 0.05, 95% CI: 0.03–0.11), neoadjuvant (RRR = 0.34, 95% CI: 0.21–0.57) or adjuvant treatment (RRR = 0.36, 95% CI: 0.22–0.59) compared to curative treatment (Table 2). For those receiving palliative treatment, there was a significant increase in 1st and 2nd line treatments from 2019 to 2020 (68% vs. 81%; *p* < 0.0001) and reduction in 3rd and subsequent lines (26% vs. 15%; *p* = 0.004) (Table 1).

### 3.3. Patients with COVID-19 Disease

Of the 2020 cohort, 42 (2%) patients developed SARS-CoV2 infections; 38% gastrointestinal, 26% breast and 17% uro-gynaecological (Table 3). The majority of these patients were on chemotherapy (64%) whilst 17% were on biological/targeted treatments. Moreover, 67% of these patients were on palliative treatment, whilst 31% were on neo/adjuvant treatment and 2% on radical. Of the 42 patients who developed COVID-19, 24 (57%) had severe infections and 6 (14%) resulted in COVID-related death.

### 3.4. Mortality

Of the 2019 cohort, 199 (8%) patients died within 6 months compared to 89 (4%) in 2020 (Table 1). In logistic regression models, the relative risk of 6-month mortality for patients treated in 2020 was statistically significantly lower than for patients treated in 2019 (OR = 0.39, 95% CI: 0.31–0.50) (Table 4). In the sensitivity analyses with only stage IV patients, stratification by line of palliative treatment did not significantly alter the results (Table 4). For those undergoing line 1 palliative treatment OR = 0.23, 95% CI: 0.16–0.36; line 2 palliative treatment OR = 0.36, 95% CI: 0.22–0.59).

## 4. Discussion

In our cohort, the delivery of SACT during the first wave of the COVID-19 pandemic did not appear to be detrimental to patients with solid tumours with low infection and death rates. The proportion of patients being treated with SACT in 2020 was lower than the comparative period in 2019 with an increase in the proportion of patients being treated in the metastatic setting. During the pandemic in 2020, the proportion of radical and first line SACT increased compared to the same period in 2019. The modality of SACT was altered in 2020 with increased use of immunotherapy and combination biological/targeted therapy and, less chemotherapy when compared to 2019. To our knowledge, this is the first published data truly identifying the risk of SACT in a defined, unselected, population with comparable pre-pandemic data from an apex comprehensive UK Cancer Centre.

Comparing the proportion of tumour types between the two time periods, the biggest changes seemed to be in GI (3% decrease −23.1% vs. 19.7%) and lung (1.5% decrease—13.0% vs. 11.5%) cancer cases on SACT during the pandemic. It is difficult to be certain of the reason for this. However, we postulate a reduced rate of SACT in these tumour groups as these patients tended to be highly symptomatic, with multiple comorbidities making them more borderline eligible for treatment and vulnerable to COVID-19 morbidity and mortality. Therefore, possibly there was a higher rate of deferral or no treatment in these tumour types. In line with an increase in metastatic disease, there appeared to be an increased reduction in patients with stage 3 disease on SACT (4.1% decrease—18.3% vs. 14.2%). Again, we believe this is likely due to deferral of treatment in this cohort at the onset of the pandemic. In comparison to those with metastatic disease where the urgency to treat would have outweighed risks of SARS-CoV2 infection and therefore treatment would have continued even in the first wave.

The odds of 6-month mortality for patients treated in 2020 was lower than for patients treated in 2019. This may have been as a result of selection bias through the clinical teams choosing to treat patients who were less frail and able to attend the hospital setting to receive their SACT. Consequently, the frailer patients with a higher risk of death would not have been captured in our data for this study. The mortality rate in the COVID positive group was similar to outcomes seen in all our oncology patients, including those not on immunosuppressive treatments [2]. Other studies have also described the safe delivery of anti-cancer treatment over the COVID-19 pandemic. For example, a nationwide collaboration in the Netherlands included 351 COVID-19 positive cancer patients of which 165 were on active treatment [3]. In this study, the authors concluded that treatment type was not associated with the risk of fatal outcome thus complementing the results from our current study [3]. Additionally, a national French study concluded that cytotoxic treatment (immunotherapy, targeted therapy and hormone therapy) within 3 months of COVID-19 diagnosis did not affect risk of all-cause mortality [4]. In a meta-analysis by Yekedüz et al. including data from sixteen studies, there was no significant difference between risk of severe COVID-19 in the chemotherapy (30-days prior to COVID-19 disease) vs. control group when adjusting for appropriate confounders [5]. However, there was an increased risk of death in the chemotherapy group compared to the control group (OR: 1.85, 95% CI: 1.26–2.71). In the same study, there was no significant increased risk of severe COVID-19 or death in the immunotherapy, targeted therapy, radiotherapy, cancer surgery or cancer treatment groups (latter four were univariate analyses only).

The COVID-19 pandemic has forced hospitals around the world to make difficult decisions regarding the continuation of care for cancer patients. In a recent survey completed by health care professionals working across 17 countries, all institutions implemented some sort of change into the delivery of anti-cancer treatment whether this be treatment delays, prioritisations or using less immunosuppressive drugs [6]. In our study, it was noted that there was an increase in the proportion of patients with stage IV disease being treated in 2020 when compared to 2019. We postulate that these numbers reflect the backlog of undiagnosed cancer cases early on in the pandemic in 2020 [7]. Furthermore, there was a lower proportion of patients receiving chemotherapy in 2020 when compared to 2019. This is indicative of the modification of SACT usage due to the COVID-19 pandemic as suggested in the survey by Chow et al. [6]. Clinicians had to weigh up the risks of patients coming into hospital to receive chemotherapy and hence it was deemed more appropriate and safer for some patients to receive alternative treatment options with a less myelosuppressive profile. There was a slight increase in the use of immunotherapy in 2020 when compared to 2019, although this result was not significant. This may reflect a rise in licensed options for immunotherapy as well as pandemic-related changes in NHS England guidance which advocated the use of immunotherapy over chemotherapy.

It has repeatedly been shown in studies (including that of our own) that patients of certain ethnicities i.e., Black and Asian are at an increased risk of both COVID-19 infection and COVID-19-related death [2,8,9]. Therefore, it is reassuring to see from our data in this current study that despite a large proportion (>20%) of Black or Asian ethnicity that the COVID-19 infection and mortality rate remained low. Though the overall mortality percentages appear to differ between 2019 and 2020, these results were not found to be statistically significant. Having said this, the lower proportion of patients dying in 2020 may be reflected by the impact of shielding, reduced neutropenic sepsis or the usage of lower doses of chemotherapy.

This study benefitted from the data from a defined population at one of the largest comprehensive cancer centres in the UK. Data were available for both wave one of the pandemic in 2020 as well as pre-pandemic data for the same time period in 2019. Despite this being single centre data, the quantity of patients seen at Guy’s hospital makes for meaningful results. The use of real world data is also a huge advantage as we see a real world example of the distribution of SES levels and ethnicities seen within London and across the UK. There are however limitations with this study including the lack of data available on the dose of SACT received by patients to investigate dose reduction and use of granulocyte stimulating factors during the first wave of the COVID-19 pandemic. The data were collected from our hospital SACT electronic prescribing system, so it might not have captured patients on hormonal treatments (for example for breast and prostate cancer) administered in primary care. Moreover, a small number of COVID-19 positive patients from peripheral hospitals could have been missed, although we feel this was minimal due to central COVID-19 testing at GSTT for all South-East London patients at that time and utilising data from our network hospitals. Finally, we only included patients with solid tumours within this study which may have influenced the low infection levels, given the haematological cancer patients are known to be at an increased risk of COVID-19 [8,10,11]. We are also aware that the use of three months’ worth of data per year may not capture all trends in stage distributions or treatment choices outside of these time periods. Furthermore, many collaborative studies are biased by the collection of COVID-19 positive cases only and are lacking in the COVID-19 negative controls which this study benefits from.

## 5. Conclusions

Data from this large comprehensive cancer centre at the epicentre of the UK COVID-19 epidemic have demonstrated that the delivery of SACT during the first wave of the COVID-19 pandemic was possible without significantly compromising mortality within 6-months of treatment. Despite the immense challenges faced by clinicians at the beginning of the pandemic, SACT delivery remained a crucial part of cancer treatment and this study has established that despite the challenges faced, SACT was able to be continued without any significant effects on the mortality of patients. The ever emerging information on how COVID-19 has affected cancer patients, such as an increase in patients with delayed diagnoses, highlights the need to find a way to continue to treat patients for their cancer even whilst the pandemic is ongoing. This data led to more confident decisions about the continuation of SACT during the second and the third wave of the COVID-19 pandemic in our institution (and could be used as guidance in national and international level).

## Figures and Tables

**Table 1:** Cohort characteristics when stratified by year of treatment.

SACT Patients	SACT		SACT	
(*n* = 4569)	2019		2020	
	(*n* = 2449)		(*n* = 2120)	
	*n*	%	*n*	%
Sex				
Male	1122	45.80	933	44.00
Female	1327	54.20	1187	56.00
Age				
<50	436	17.80	387	18.30
50–59	541	22.10	499	23.50
60–69	683	27.90	607	28.60
70–79	603	24.60	477	22.50
≥80	186	7.60	150	7.10
Mean (SD)	62.50 (13.20)		61.80 (13.00)	
SES				
Low	2043	83.40	1804	85.10
Medium	69	2.80	41	1.90
High	246	10.00	185	8.70
Missing	91	3.70	90	4.20
Ethnicity				
White British	1171	47.80	831	39.20
White Other	191	7.80	172	8.10
Black Caribbean	117	4.80	74	3.50
Black African	94	3.80	96	4.50
Black Other	56	2.30	50	2.40
Asian	64	2.60	51	2.40
Mixed	30	1.20	23	1.10
Other	66	2.70	43	2.00
Unknown	660	26.90	780	36.80
Tumour Type				
Urological	534	21.80	456	21.50
Gynaecological	185	7.60	174	8.20
Gastrointestinal	565	23.10	418	19.70
Skin/Head and Neck	153	6.20	142	6.70
CNS	93	3.80	62	2.90
Breast	595	24.30	571	26.90
Lung	281	11.50	275	13.00
Other	43	1.80	22	1.00
Stage				
1	119	4.90	69	3.30
2	242	9.90	186	8.80
3	448	18.30	301	14.20
4	1529	62.40	1527	72.00
Missing	111	4.50	37	1.70
SACT Type				
Chemotherapy	1027	41.90	725	34.20
Immunotherapy	157	6.40	208	9.80
Biological/Targeted	911	37.20	806	38.00
Combo Biological/Targeted	126	5.10	210	9.90
Combined Chemo	206	8.40	145	6.80
Combined Immunotherapy	22	0.90	26	1.20
Treatment Paradigm				
Neoadjuvant	203	8.30	176	8.30
Adjuvant	503	20.50	418	19.70
Radical	100	4.10	14	0.70
Palliative	1630	66.60	1453	68.50
Curative	13	0.50	59	2.80
Line of Palliative Treatment (2019, *n* = 1630; 2020, *n* = 1453)				
0	2	0.10	1	0.10
1	594	36.40	678	46.70
2	512	31.40	497	34.20
3	268	16.40	130	9.00
4	110	6.80	51	3.50
5	50	3.10	29	2.00
Missing	94	5.80	67	4.60
Overall mortality at 6 months				
	317	12.90	123	5.80

SES—socioeconomic status.

**Table 2:** Relative risk ratios for treatment type, treatment paradigm and cancer stage between paTable 2019. versus 2020.

Variable	RRR ^a^	95% Confidence Interval
SACT type		
Chemotherapy	(Base outcome)	
Immunotherapy		
2019	1.00	Ref.
2020	1.69	(1.35–2.11)
Biological/Targeted		
2019	1.00	Ref.
2020	1.27	(1.11–1.45)
Combination Biological/Targeted		
2019	1.00	Ref.
2020	2.30	(1.82–2.92)
Combined chemotherapy		
2019	1.00	Ref.
2020	0.99	(0.77–1.24)
Combined immunotherapy		
2019	1.00	Ref.
2020	1.30	(0.76–2.23)
Treatment Paradigm		
Curative	(Base outcome)	
Neoadjuvant		
2019	1.00	Ref.
2020	0.34	(0.21–0.57)
Adjuvant		
2019	1.00	Ref.
2020	0.36	(0.22–0.59)
Radical		
2019	1.00	Ref.
2020	0.05	(0.03–0.11)
Palliative		
2019	1.00	Ref.
2020	0.39	(0.24–0.62)
Cancer stage		
Stage I	(Base outcome)	
Stage II		
2019	1.00	Ref.
2020	1.44	(1.01–2.05)
Stage III		
2019	1.00	Ref.
2020	1.11	(0.80–1.54)
Stage IV		
2019	1.00	Ref.
2020	1.89	(1.40–2.57)

RRR ^a^—Adjusted for age, sex, ethnicity and socioeconomic status.

**Table 3:** Characteristics of COVID-19 positive patients when stratified by tumour type.

SACT 2020	All		Urological		Gynaecological		GI		Skin/Head and Neck		Breast		Lung	
COVID-19 Positive	*N* = 42		*N* = 6		*N* = 1		*N* = 16		*N* = 2		*N* = 11		*N* = 6	
	*n*	%	*n*	%	*n*	%	*n*	%	*n*	%	*n*	%	*n*	%
Sex														
Male	18	42.90	6	100	0	0.00	9	56.30	1	50.00	0	0.00	2	33.30
Female	24	57.10	0	0.00	1	100	7	43.80	1	50.00	11	100	4	66.70
Age														
<50	9	21.40	1	16.70	1	100	4	25.00	0	0.00	2	18.20	1	16.70
50–59	11	26.20	0	0.00	0	0.00	5	31.30	0	0.00	4	36.40	2	33.30
60–69	13	31.00	4	66.70	0	0.00	4	25.00	1	50.00	2	18.20	2	33.30
70–79	8	19.00	1	16.70	0	0.00	3	18.80	0	0.00	3	27.30	1	16.70
≥80	1	2.40	0	0.00	0	0.00	0	0.00	1	50.00	0	0.00	0	0.00
Mean (SD)	59.30 (11.90)		60.40 (13.00)		48.70 (N/A)		57.50 (11.90)		74.20 (15.50)		59.30 (12.70)		59.50 (8.80)	
SES														
Low	38	90.50	5	83.30	1	100	15	93.80	1	50.00	10	90.90	6	100
Medium														
High	2	4.80	0	0.00	0	0.00	0	0.00	1	50.00	1	9.10	0	0.00
Missing	2	4.80	1	16.70	0	0.00	1	6.30	0	0.00	0	0.00	0	0.00
Ethnicity														
White British	13	31.00	3	50.00	0	0.00	4	25.00	0	0.00	3	27.30	3	50.00
White Other	6	14.30	0	0.00	0	0.00	2	12.50	1	50.00	2	18.20	1	16.70
Black Caribbean	1	2.40	0	0.00	0	0.00	1	6.30	0	0.00	0	0.00	0	0.00
Black African	6	14.30	1	16.70	0	0.00	2	12.50	0	0.00	3	27.30	0	0.00
Black Other	3	7.10	0	0.00	0	0.00	1	6.30	0	0.00	1	9.10	1	16.70
Asian	0	0.00	0	0.00	0	0.00	0	0.00	0	0.00	0	0.00	0	0.00
Mixed	0	0.00	0	0.00	0	0.00	0	0.00	0	0.00	0	0.00	0	0.00
Other	1	2.40	1	16.70	0	0.00	0	0.00	0	0.00	0	0.00	0	0.00
Unknown	12	28.60	1	16.70	1	100	6	37.50	1	50.00	2	18.20	1	16.70
Comorbidities														
Hypertension	15	35.70	3	50.00	1	100	6	37.50	2	100	2	18.20	1	16.70
Diabetes Mellitus	9	21.40	3	50.00	0	0.00	2	12.50	1	50.00	2	18.20	1	16.70
Lung Conditions	5	11.90	0	0.00	0	0.00	1	6.30	0	0.00	2	18.20	2	33.30
Renal Impairment	2	4.80	1	16.70	0	0.00	1	6.30	0	0.00	0	0.00	0	0.00
Liver Conditions	1	2.40	0	0.00	0	0.00	0	0.00	0	0.00	0	0.00	1	16.70
CVD	2	4.80	0	0.00	0	0.00	1	6.30	1	50.00	0	0.00	0	0.00
Frailty	3	7.10	1	16.70	0	0.00	0	0.00	0	0.00	1	9.10	1	16.70
Chronic Steroid Use	0	0.00	0	0.00	0	0.00	0	0.00	0	0.00	0	0.00	0	0.00
No. of Comorbidities														
0	20	47.60	3	50.00	0	0.00	8	50.00	0	0.00	7	63.60	2	33.30
1	13	31.00	0	0.00	1	100	6	37.50	1	50.00	2	18.20	3	50.00
2	3	7.10	1	16.70	0	0.00	1	6.30	0	0.00	1	9.10	0	0.00
3+	6	14.30	2	33.30	0	0.00	1	6.30	1	50.00	1	9.10	1	16.70
Smoking history														
Never	19	45.20	2	33.30	0	0.00	7	43.80	1	50.00	7	63.60	2	33.30
Current	2	4.80	0	0.00	0	0.00	1	6.30	0	0.00	0	0.00	1	16.70
Ex-smoker	9	21.40	2	33.30	0	0.00	4	25.00	0	0.00	1	9.10	2	33.30
Unknown	12	28.60	2	33.30	1	100	4	25.00	1	50.00	3	27.30	1	16.70
SACT														
Chemotherapy	27	64.30	1	16.70	1	100	14	87.50	1	50.00	9	81.80	1	16.70
Immunotherapy	4	9.50	1	16.70	0	0.00	0	0.00	0	0.00	0	0.00	3	50.00
Biological/Targeted	7	16.70	4	66.70	0	0.00	0	0.00	1	50.00	1	9.10	1	16.70
Combo Biological/Targeted	2	4.80	0	0.00	0	0.00	1	6.30	0	0.00	0	0.00	1	16.70
Combined Chemo	2	4.80	0	0.00	0	0.00	1	6.30	0	0.00	1	9.10	0	0.00
Treatment Paradigm														
Neoadjuvant	8	19.00	0	0.00	0	0.00	4	25.00	0	0.00	4	36.40	0	0.00
Adjuvant	5	11.90	0	0.00	0	0.00	4	25.00	0	0.00	0	0.00	1	16.70
Radical	1	2.40	1	16.70	0	0.00	0	0.00	0	0.00	0	0.00	0	0.00
Palliative	28	66.70	5	83.30	1	100	8	50.00	2	100	7	63.60	5	83.30
COVID severity														
Mild	10	23.80	0	0.00	0	0.00	7	43.80	0	0.00	1	9.10	2	33.30
Pneumonia	2	4.80	0	0.00	0	0.00	2	12.50	0	0.00	0	0.00	0	0.00
Severe pneumonia	24	57.10	5	83.30	1	100	4	25.00	2	100	9	81.80	3	50.00
COVID-related death	6	14.30	1	16.70	0	0.00	3	18.80	0	0.00	1	9.10	1	16.70

SES—socioeconomic status; CVD—cardiovascular disease; NSAID—non-steroidal anti-inflammatory drugs; ACE—angiotensin-converting enzyme; ARB—angiotensin receptor blockers.

**Table 4:** Logistic regression analysis for risk of death within 6-months of treatment.

Variable	OR ^a^	95% CI
Year		
2019	1.00	Ref.
2020	0.39 *	(0.31–0.50)
Sensitivity Analyses:		
Stage IV, Line 1 palliative treatment		
Year		
2019	1.00	Ref.
2020	0.23 **	(0.16–0.36)
Stage IV, Line 2 palliative treatment		
Year		
2019	1.00	Ref.
2020	0.36 **	(0.22–0.59)

OR ^a^—Adjusted odds ratio. * Adjusted for age, sex, ethnicity, socioeconomic status, cancer type, cancer stage and treatment paradigm. ** Adjusted for age, sex, ethnicity, socioeconomic status and cancer type.

## Data Availability

The data presented in this study are available on request from the corresponding author. The data are not publicly available due to ethical reasons.
